# Hyperspectral image and chemometrics. A step beyond classical spectroscopic PAT tools

**DOI:** 10.1007/s00216-025-06154-x

**Published:** 2025-10-28

**Authors:** Anna de Juan, Rodrigo Rocha de Oliveira

**Affiliations:** Chemometrics Group, Department of Chemical Engineering and Analytical Chemistry, Universitat de Barcelona, Martí I Franquès, 1, 08028 Barcelona, Spain

**Keywords:** Hyperspectral imaging, Process analytical technology (PAT), Soft sensors, Process chemometrics

## Abstract

Hyperspectral imaging (HSI) is a very complete analytical measurement that encloses rich spatial and chemical information. This double side enables HSI to outperform classical spectroscopic measurements and vision systems based only on color information. However, HSI requires powerful data analysis tools for interpretation and to facilitate its implementation in process analytical technology (PAT) contexts. This work is a brief perspective that shows a diversity of PAT challenges that can be solved with the combined use of HSI and dedicated chemometric procedures.

## Introduction

The use of spectroscopy as an essential process analytical technology (PAT) tool dates from several decades. Indeed, the progress in the development of smaller, robust, and affordable sensors has been key in the implementation of PAT in many contexts of industrial routine monitoring and process control. Spectroscopic sensors offer the advantage of providing complementary information about the chemistry and composition of systems depending on the technique chosen. Moreover, the versatility of the devices adapts to off-line, at-line, and in-line monitoring setups [1–3]. However, the intrinsic spectroscopic measure would lack applicability if it were not connected with powerful data analysis (chemometrics) techniques that allow embedding models that can transform the spectroscopic input into qualitative and quantitative information. These models are called soft sensors and enable automated real-time decision-making tasks in a process context [4–6]. Spectroscopic sensors can be designed to perform measurements in solid, liquid, or gas samples and provide information about a specific sampling spot, which can vary in spatial scale depending on the field of view of the sensor and the spectroscopic technique used.

When dealing with solid samples and high throughput production, observation of a single or a few sampling spots may not be sufficient to characterize a material or an object. In these scenarios, the introduction of more detailed spatial information is mandatory. To assist in this need, PAT has incorporated vision systems that provide a complete picture on samples and materials in static mode or in moving streams at different spatial scales. Vision systems can be extremely helpful in tasks such as product sorting in waste moving streams or defect detection in food products, to mention a few known examples [7–10]. For speed and affordability, the most usual vision systems are based on the measurement of color. Indeed, this simple information is often sufficient and, when treated appropriately, can also provide indirect information about texture and object shape. There are many examples where the proper extraction and use of color, texture, and/or shape features associated with images can provide clear answers in a PAT context [11]. However, these simple vision systems do not provide answers about chemical (composition) and physical (polymorphism) properties of samples, which are of utmost importance for some applications.

Hyperspectral images (HSI) appear as the PAT tool able to link the chemical information and the spatial description of samples. HSIs are obtained by investigating a large sample area and associating a full spectrum with every pixel scanned. In this way, all benefits linked to spectroscopic sensors and vision systems are found in a single measurement. Nowadays, imaging systems are coupled to a large variety of spectroscopies, ranging from IR, Raman, and UV spectroscopies to fluorescence and mass spectrometry, to mention some of the most used. The spectroscopic wavelength range and the requirements for spectroscopic acquisition determine the spatial resolution of the image, making HSI a measurement that can cover a large span of multispectroscopic and multiscale scenarios [12–17].

The different image acquisition modes point towards specific applications. Thus, point-scanning acquisition that works by acquiring one pixel spectrum at a time is more suitable when an off-line accurate description of product properties needs to be carried out. Instead, the line-scanning mode operates by acquiring simultaneously a line of pixel spectra on a material moving stream. The frame rate of acquisition in these systems can be extremely fast, and they are ideal for real-time on-line monitoring [18–22].

If classical spectroscopic devices were coupled with soft sensors, this requirement is even more crucial to interpret the HSI measurement. At present, all the chemometric tools devoted to performing exploration, calibration, classification, unmixing, or process control tasks on spectroscopic measurements are adapted to handle image data, although there is still room for improvement in this respect. A specific aspect of imaging is that the output of soft sensors can be provided at different levels, i.e., at an image level, when a single answer refers to the full area scanned, e.g., global composition information; at an object level, when the image is formed by different spatially separated units, e.g., composition provided for every single fruit or pill unit in an image; or at a pixel level, when the same information relates specifically to every pixel scanned. Very often, the same soft sensor (model) is able to provide output information at all levels required. In general, the nature of the problem determines which level can be more suitable for interpretation purposes [13, 23, 24].

The following sections are focused on providing a brief overview of the potential of different soft sensors on imaging data for industrial applications. Rather than being an exhaustive review, the idea is to show diverse and representative examples of the joint use of HSI and chemometrics to promote the use of this technology.

## HSI data structure

**Fig. 1:**
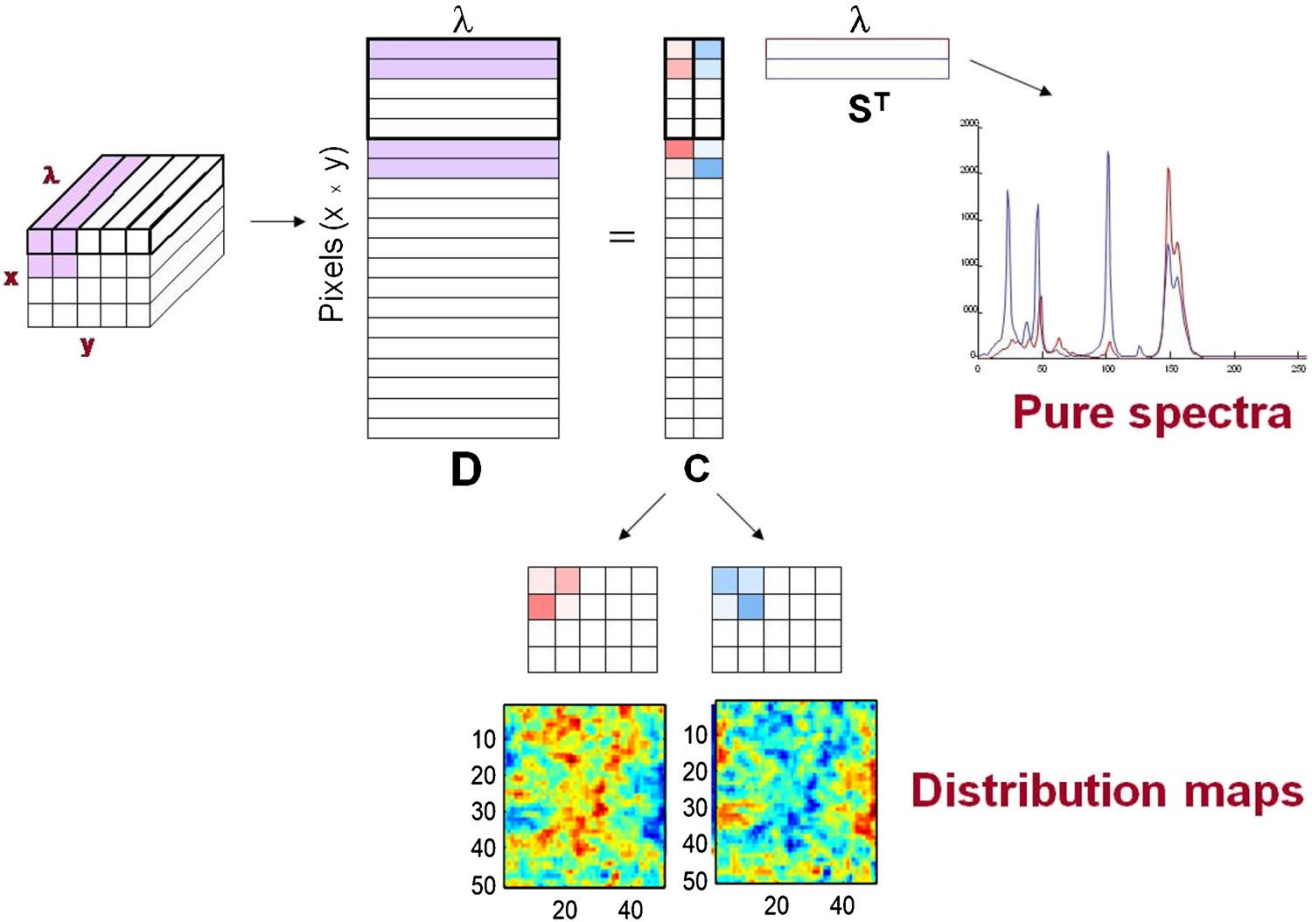
Interpretation of the information of an image data cube as a Beer-Lambert-like bilinear model formed by the spectral signatures and related concentration profiles of the image constituents. Concentration profiles are refolded into concentration maps to recover spatial information

A HSI can be described as a data cube, with two spatial dimensions (pixel coordinates) and a spectral dimension (see Fig. 1). Although this representation covers the majority of imaging measurements, there may be examples of images with three spatial dimensions, e.g., multilayer acquisitions on the same sample, or images collecting 2D spectroscopic signals, e.g., fluorescence images where every pixel is connected to a full 2D excitation-emission landscape. Despite the image cube structure in Fig. 1, it is important to point out that most chemometric methods treat images as an ensemble of spectra structured as a data table [12, 13, 24]. The reason is that, with suitable preprocessing, the spectroscopic measurement obeys a bilinear model similar to the Beer-Lambert law, as shown in Eq. 1,

1 $$\mathrm D=\mathrm{CS}^{\mathrm T}+\mathrm E$$

where the table of initial spectroscopic raw measurement (**D**) can be described as the combination of the spectral signatures of the pure image constituents (**S**^**T**^) weighted by their concentration in the different pixels (**C**). If spatial information needs to be recovered, it is sufficient to refold the pixel concentration profiles of every image constituent into the original 2D spatial geometry of the image to obtain the concentration maps.

The linear model in Eq. 1 is not only the basis of image unmixing methodologies, which provide in a straightforward way the maps and spectral signatures of image constituents from the raw images, but also the reason why many linear chemometric methods used in spectroscopy for exploratory, calibration, classification, or process control purposes can easily adapt to the hyperspectral image context, as will be seen in the following sections. For instance, principal component analysis (PCA), the most widely known linear methodology in chemometrics, has played, and continues to play, a fundamental role in multivariate image analysis [25, 26]. Beyond its classical use in exploratory analysis and dimensionality reduction, PCA-based control charts, e.g., using Hotelling’s *T*^2^ and *Q*-residuals statistics, constructed from hyperspectral images provide an efficient framework for multivariate statistical process monitoring and process control in PAT contexts [27]. Even if this is not the main focus of this work, we encourage the reader to consult dedicated reviews for a comprehensive overview of the use of PCA and related methods in process industries [28, 29].

Therefore, to conclude this section, we need to consider that although the use of deep learning methodologies for hyperspectral image processing [30] is a hype and may find justification in specific contexts, we should not forget that the nature of the HSI measurement is linear and, hence, linear methods provide simpler and robust data pipelines, less computationally intensive, and should be generally the first choice.

## HSI and quantitative information

The transformation of a spectroscopic input into one or more quantitative information outputs has been the task carried out with soft sensors embedding multivariate calibration models based on partial least squares regression or other methodologies. As mentioned in the “Introduction,” the use of classical spectroscopic sensors for this purpose is in the routine of many industrial processes to extract quantitative information in real time. However, the narrow field of view of these devices compromises the representativeness of the results obtained. HSIs come then into play.

**Fig. 2:**
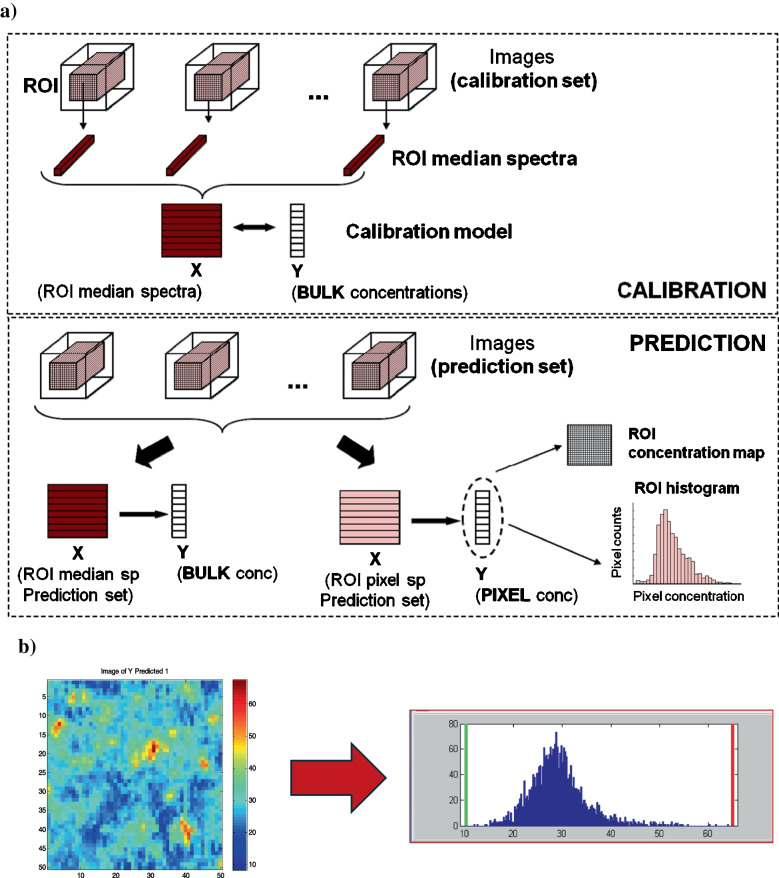
Use of calibration in an HSI context. **a** Generation of a calibration model using the median spectra of several calibration images. Prediction performed at a global image level (from median spectra) or at a pixel level. **b** Example of a predicted concentration map of a sample containing 30% caffein. Related histogram based on pixel concentration values

The adaptation of calibration tools to imaging starts by building off-line calibration models, which take as spectral information the mean or the median spectrum of calibration images to be related to the bulk quantitative information of the samples scanned (see Fig. 2). This step does not differ from the construction of calibration models using classical spectroscopic sensors.

The main difference lies in the use of the calibration models for prediction. In this context, the quantitative prediction can be carried out at an image level, i.e., using the mean or median spectrum of new images as input information to obtain bulk image concentration values or, more interestingly, at a pixel level [13, 24].

Quantitative predictions at a pixel level allow for obtaining concentration maps that clearly show other aspects connected with the spatial information, such as the presence of composition trends across the sample space, e.g., for certain food constituents [22, 31–34]. Indeed, food quality is no longer defined by a single quantitative composition parameter per constituent, but rather by the distribution across the sample surface of fat, moisture, or other relevant components. The measurement of moisture is related to important food quality attributes, and NIR imaging is highly suitable to determine this parameter. Thus, Kamruzzaman et al. [32] used the moisture determination and spatial distribution to determine the water holding capacity in meat, a parameter for which high values are desirable since they indicate a low drip loss when meat is cooked. He et al. [33] also used the determination and distribution of moisture in salmon fillets as a quality indicator. Likewise, many food products undergo crucial transformations, e.g., cheese ripening, where it is essential to have a quantitative spatial mapping of dehydration, proteolysis, and lipolysis during the process [31].

The pharmaceutical field and, in general, any industrial application that involves the mixture of compounds to elaborate a final product requires a methodology that provides a good quantitative spatial description of the heterogeneity, an essential quality attribute in this context [35, 36]. The definition of heterogeneity takes as seeding information the pixel concentration values of the maps to build histograms or to derive more complex heterogeneity indicators based on approaches such as macropixel or variographic analysis [16, 35–38]. More on this aspect will be described in section “HSI and process understanding” linked to process understanding.

It is important to note that the use of soft calibration sensors for imaging allows working off-line, taking the information of individual images previously recorded [16, 36–38], or on-line in a line-scanning context by predicting simultaneously the concentration values linked to each line of spectra in real-time [32, 33].

## HSI and qualitative information

There are many PAT contexts where the image input is expected to provide qualitative information. For instance, in a sorting problem, the question may be whether an object is made of a certain material or not [23], or in a cereal moving stream, the interest may be detecting whether strange objects are present or not [39]. For all these questions, a positive answer needs to be linked with a location and, hence, the imaging measurement fits perfectly the purpose.

The qualitative questions above or similar ones may be answered in different ways. A first approach may be based on unsupervised clustering methods that, without using prior known information, will divide (segment) all the image surface scanned into different zones according to their chemical properties; i.e., pixels will be grouped according to their spectral (chemical) similarity. This option may be valid for off-line applications when an image collected can be fully analyzed and has a first exploratory value about the complexity of the material analyzed.

However, the most common option to obtain qualitative information in PAT contexts is provided by the so-called classification models that link the hyperspectral input to predefined qualitative class membership information. In a sorting problem, every kind of material defines a class; e.g., in the cereal moving stream case, the wheat can be a class, and anything out of it and different from a background may be identified as a strange object. In a classification context, the classification model is built off-line, based on images with objects or materials with a known class attribution. Once the model is built, it can be used off- or on-line to predict class information from new image inputs.

Classification is one of the contexts where the imaging input can range from color to multispectral (formed by a few bands) and finally hyperspectral information. The complexity of the input is clearly associated with the natural attributes of the sample and the difficulty in discriminating materials or objects from different classes; e.g., vision systems may be sufficient to distinguish colored materials. Hyperspectral imaging is preserved to address problems where chemical composition is the driving force for class discrimination and the classes to be distinguished may show subtle differences among them, rendering a simpler multispectral input insufficient.

Image-based classification is one of the fields where prediction at different levels becomes particularly relevant [23]. Thus, obtaining class membership information at a pixel level is ideal to study heterogeneous materials, where knowing in detail the spatial pattern of distribution of materials may be the key question. However, working at a pixel level may not provide a clear answer when the composition of the classes is too similar or, simply, when a single answer is required per object scanned.

**Fig. 3:**
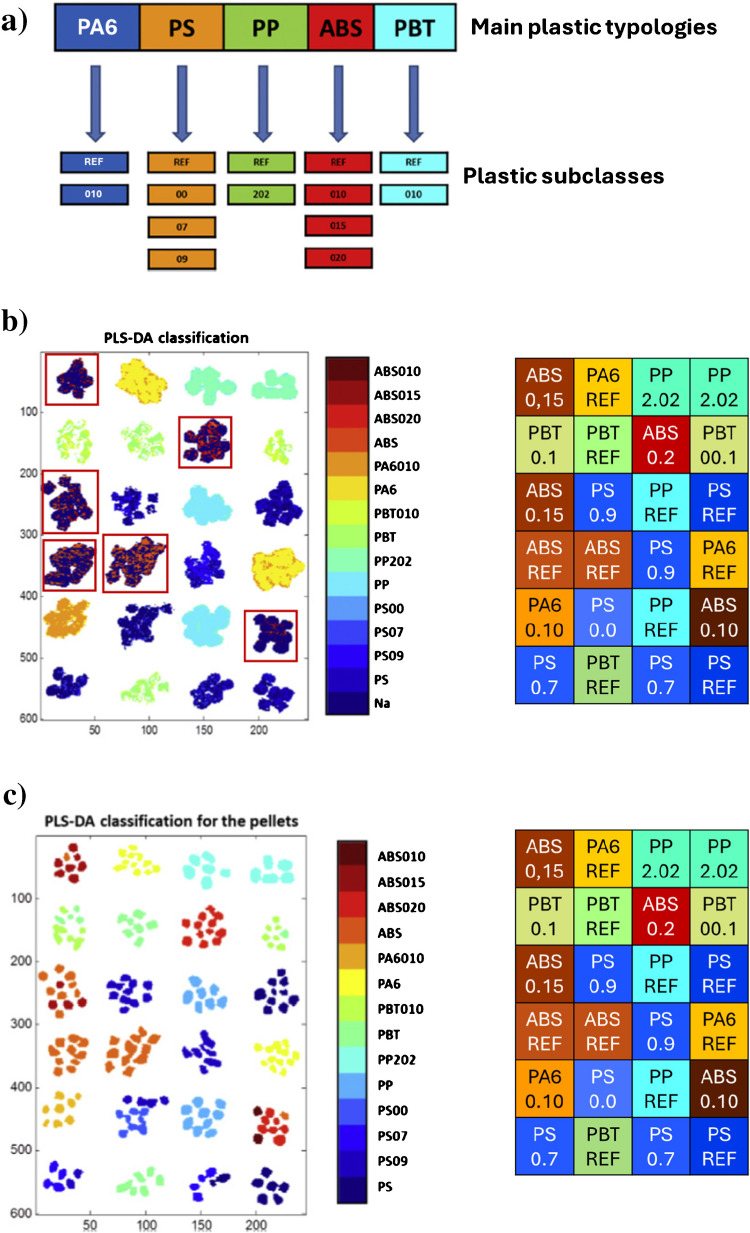
**a** Scheme of main plastic categories, acrylonitrile butadiene styrene (ABS), polyamide 6 (PA6), polybutylene terephthalate (PBT), polypropylene (PP), and polystyrene (PS) and subclasses within each plastic category, related to the main plastic categories with different amounts of flame retardants added (for more information, see reference [23]). **b** Classification model results for plastic subclass identification based on a pixel level approach. Right plot: ground truth for location and color code for plastic pellet subclasses. Left plot: plastic pellets colored according to predicted class. Many pixels are misclassified or do not have a clear class assigned (dark blue pixels within red squares). **c** Classification model results for plastic subclass identification based on an object-level approach. Meaning of left and right plot as in **b**. Almost all plastic pellets are correctly attributed to their class

**Fig. 4:**
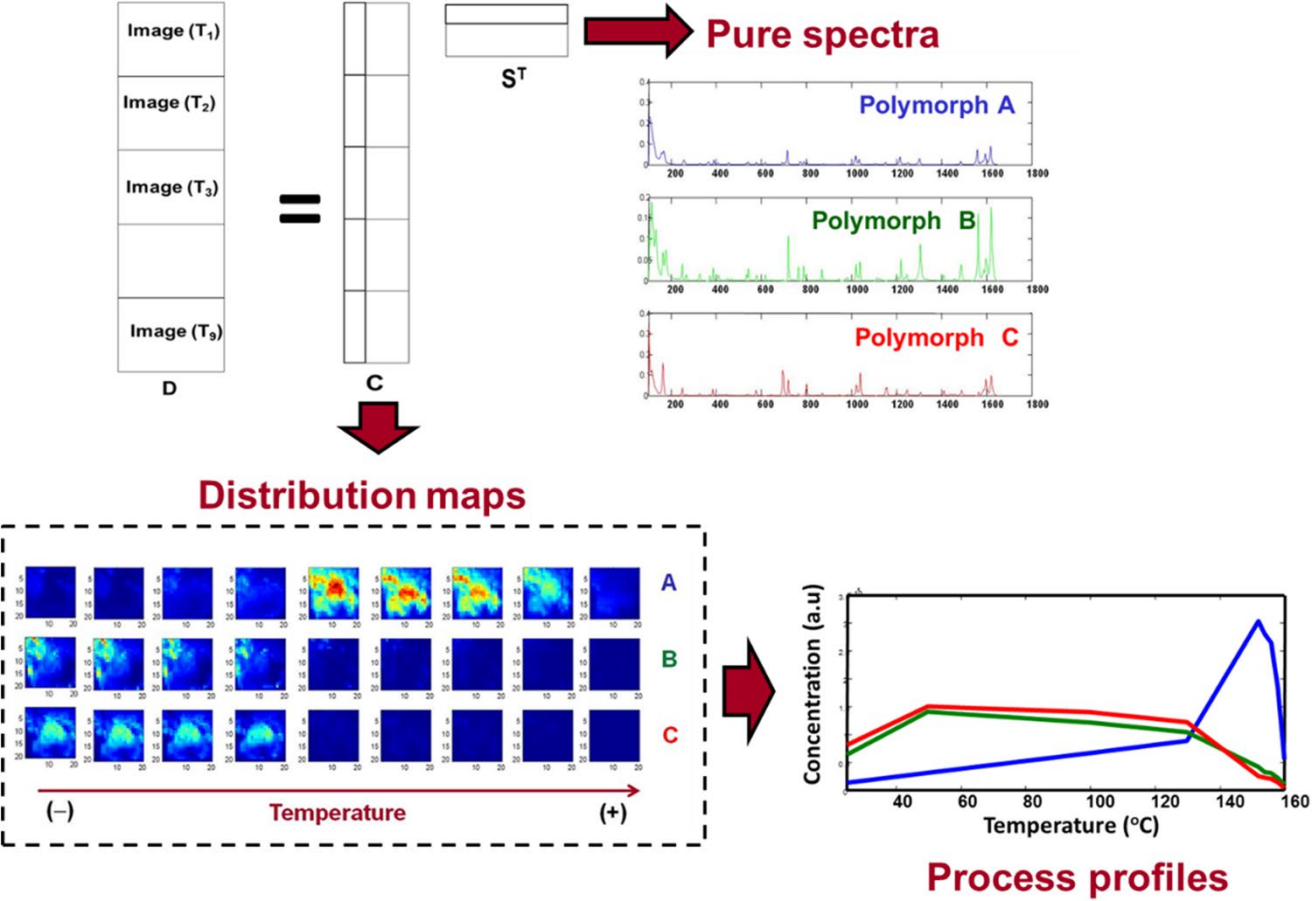
Study of a polymorphic thermal transition based on Raman imaging and use of MCR. Image multiset structure formed by image blocks of pixel spectra collected at different temperatures. Right hand upper plot: pure spectral Raman fingerprints of the polymorphs involved. Down left plot: evolution of concentration maps with temperature. Down right plot: thermal-dependent polymorph profiles obtained from the average values of the concentration maps

A very illustrative example is provided by Amigo et al. in a microplastic sorting problem [23]. The goal of the study was to separate microplastic particles per plastic typologies in two steps: a first step, where five categories of very different plastics had to be distinguished (identified in Fig. 4a as PA6, PP, PS, ABS, and PT), and a second step, where subclasses within each main plastic category had to be further differentiated (branches below each main plastic category in Fig. 4a, which were related to the same basic plastic type with different amounts of flame retardants added). In the first step, working at a pixel level was sufficient to unequivocally identify the main plastic typology of every pellet since a vast majority of pixels within a particle were assigned to the same class (not shown; for results, see [23]). In the second step, when classification models were aimed at plastic subclass detection, two strategies were tried, namely working at a pixel level (Fig. 4b) and at an object level (Fig. 4c). Working at a pixel level provided poorer results because of the spectral similarity among classes (see that many pixels were not identified with their correct class color code). In this context, a clear improvement in the classification was obtained by working at an object level (Fig. 4c), where the classification answer was based on the input of the average object spectrum (extracted from all spectra forming an individual plastic pellet). In this instance, working with the average spectrum increased the signal-to-noise ratio, decreased the influence of extreme pixel spectra, and improved the capacity to distinguish among objects of similar classes (Fig. 3).

## HSI and process understanding

The use of hyperspectral imaging responds to the need of understanding the variation of composition of materials across space. This variation of composition implies identifying the kind of materials, quantifying them, and getting to know their spatial distribution. The answer to all these aspects is straightforwardly provided by the so-called image unmixing methods. Indeed, these methodologies are based on Eq. 1 and provide the qualitative, quantitative, and spatial information associated with each of the image constituents in a sample. There are many unmixing algorithms, based usually on linear models when HSI systems are used in controlled illumination and image acquisition setups, i.e., in most laboratory or industrial scenarios [24], or in non-linear models in remote sensing [40]. Multivariate curve resolution (MCR) is an algorithm largely used for HSI and consists of optimizing the pure spectral fingerprints (**S** matrix) and the pixel concentration arrays (**C** matrix) of image constituents under natural constraints, e.g., non-negativity, by using only the raw image spectra as input information. Unmixing methodologies aim at providing chemically meaningful information of the image constituents, readily interpretable by the scientists or process operators [41]. The simplest use of unmixing methods can be the characterization of materials and products from an individual HSI associated with a particular platform. However, it is important to know that sets of related images can be analyzed together, e.g., when different layers of material are scanned at different depths, or when the same material is scanned using different imaging platforms in an image fusion context [42].

A particularly interesting use of image unmixing in the PAT context is image-based process monitoring. In such a case, the evolution of the process is described through the simultaneous analysis of all images acquired along the process evolution [43, 44]. A typical data arrangement consists of concatenating the blocks of pixel spectra associated with every image recorded across the process in a single structure. Figure 4 shows an illustrative example oriented to understand the thermal-dependent polymorphic transformation of a pharmaceutical product [44]. In this case, a set of images collected at different temperatures on the same sample was arranged in a single data structure. The unmixing procedure provided a model following Eq. 1, where the matrix **S** offered the fingerprints of the three polymorphic forms and the long concentration profiles in **C** linked to the different image blocks provided, after refolding, the variation of the concentration maps as a function of the temperature. These maps showed the clear decay of two polymorphic forms to favor the emergence of a third one when the temperature was increasing and enabled one to appreciate the spatial progress of the process. To complete the description, displaying the mean values of the concentration maps as a function of the temperature also furnished the thermal-dependent profiles of the polymorphic transformation.

Some processes may be straightforwardly focused on capturing the variation of the spatial distribution of materials in a particular product. It is the case of the blending monitoring, one of the most relevant industrial processes where HSI combined with chemometrics offers significant advantages [38, 45, 46]. The primary goal of blending is to achieve a homogeneous mixture of different components, which is essential for product quality in industries such as pharmaceuticals, food, and chemicals.

**Fig. 5:**
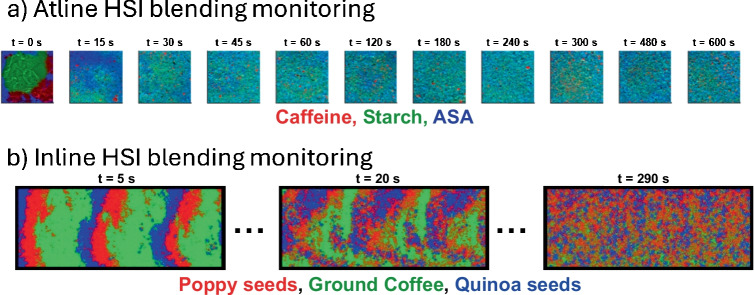
Distribution maps (RGB overlay) from **a** at-line and **b** in-line HSI blending monitoring. False color is used to display every constituent in the blending. In both **a** and **b** blending processes, a gradual loss of the segregation of the blending materials with time is clearly perceived

HSI-based blending monitoring works by continuously acquiring images during the blending process, which can be obtained at-line, i.e., stopping the process and acquiring a sample image at certain time intervals [38], or in-line, i.e., when the images are acquired in real time during the blending process [47]. These images, when subjected to unmixing analysis, provide distribution maps of each component in the mixture. The evolution of these distribution maps over time offers a comprehensive understanding of the blending dynamics, including the detection of abnormal behaviors such as de-mixing or insufficient mixing. Figure 5 shows the distribution maps obtained after unmixing analysis for the blending monitoring of a pharmaceutical formulation (Fig. 5a) and food ingredients (Fig. 5b) using an at-line and an in-line NIR-HSI monitoring approach, respectively. These distribution maps provide qualitative insight into heterogeneity, offering a direct visualization of (a) zones of segregation and layering in early stages, (b) intermediate blending progress marked by a reduction in pure compounds domain size, (c) uniform spatial distribution at later stages (indicating the potential mixing endpoint), and (d) possible de-mixing phenomena due to over-blending. Such visualization allows process analysts to intuitively assess whether materials are adequately dispersed and whether any adjustments in time, speed, or formulation are needed.

While distribution maps offer qualitative blending understanding, objective and quantitative assessment of blend uniformity is crucial. For this purpose, heterogeneity indices derived from compound-specific distribution maps have provided a statistical framework for the mixing process control.

The evaluation of heterogeneity in blending processes requires quantifying not only the overall variability in material composition but also in its spatial distribution. Based on the framework of the Theory of Sampling (TOS) [48], two dimensions of heterogeneity are emphasized: global heterogeneity (GH), which reflects the total variance in pixel concentration values across a map, and distributional heterogeneity (DH), which captures the spatial correlation or pattern of these values.

The analysis of HSI data allows both aspects to be addressed comprehensively. After generating concentration maps of each component via unmixing techniques, the maps can be assessed using variographic analysis, a method that evaluates spatial variance as a function of lag distance between pixels [38]. From this analysis, two complementary indices can be derived:

- **Global Heterogeneity Index (GHI):** Quantifies the total variance among pixels, indicating how much pixel concentration values deviate from the mean.
- **Distributional Uniformity Index (DUI):** Defines the degree of uniformity in the spatial distribution of materials, going from 0 (indicating high spatial segregation, low mixing) to 1 (uniform material distribution, good mixing).

These indices are sensitive to the spatial scale of analysis and can be tuned to probe heterogeneity at different levels of spatial scrutiny, to study from macroscopic trends to fine-grained local patterns [47].

For real-time monitoring of blending processes, line-scanning pushbroom HSI systems, especially in the NIR spectral range, have proven especially suitable for investigating continuous image streams during blending. These systems enable in situ acquisition of spatially resolved spectral data across the sample as it evolves over time. The resulting hyperspectral data cube captures both temporal and spatial heterogeneity, which is vital for understanding and optimizing mixing efficiency.

In this direction, the sliding window variographic image analysis (SWiVIA) integrates HSI with variographic analysis to continuously derive the GHI and DUI heterogeneity indices that characterize the state of blending [47]. The method involves converting hyperspectral images into compound-specific distribution maps in real time using a fast unmixing step. These maps are then evaluated using a sliding time window to generate the aforementioned heterogeneity indices.

**Fig. 6:**
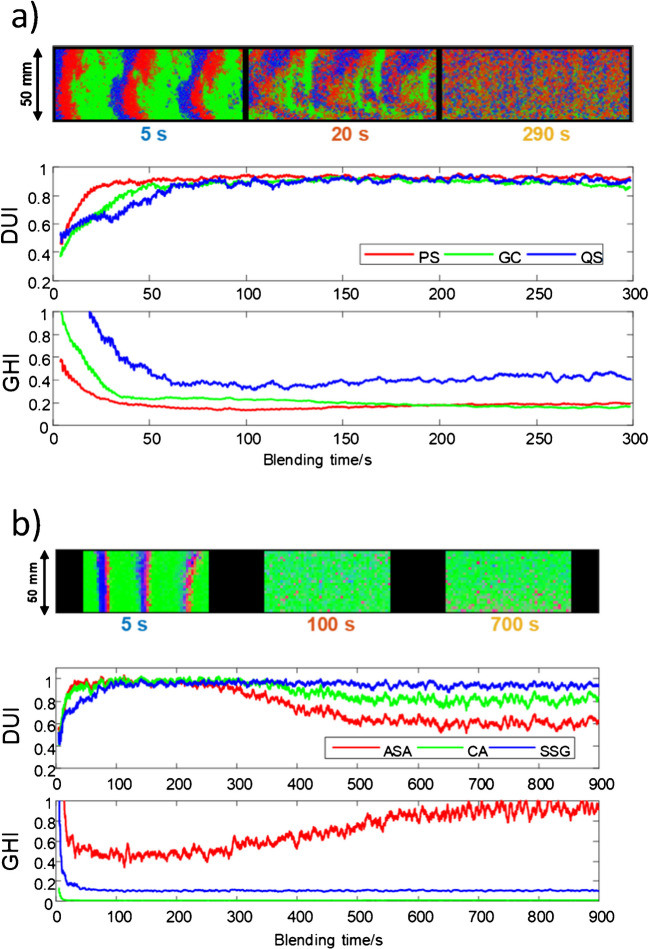
SWiVIA-derived heterogeneity curves for the continuous monitoring of food (**a**) and pharmaceutic (**b**) material blending. The top panel shows the combined RGB submaps overlaying the pure component distribution submaps for selected reference times. The mid and bottom panels show the evolution of DUI and GHI curves, respectively. **a** Mixture of poppy seeds (PS) in red, ground coffee (GC) in green, and quinoa seeds (QS) in blue. **b** Mixture of acetyl salicylic acid (ASA) in red, citric acid (CA) in green, and sodium starch glycolate (SSG) in blue. Adapted from [47]

The application of SWiVIA is illustrated in Fig. 6, which shows two blending case studies—one involving a food powder mixture (poppy seeds [PS], ground coffee [GC], quinoa seeds [QS]) and the other a pharmaceutical formulation (acetylsalicylic acid [ASA], citric acid [CA], sodium starch glycolate [SSG]). In both cases, compound-specific distribution maps and the corresponding evolution of the DUI and GHI are plotted over the blending time.

Figure 6a shows the expected correct blending behavior for the food mixture. Initially, the materials are highly segregated, as can be seen on the distribution map related to 5 s blending. Mixing progress at 20 and 290 s displays that the initially distinct regions of segregated materials gradually blend into a more uniform spatial arrangement. Accordingly, the DUI value increases steadily and reaches a plateau around 0.9, indicating that the spatial distribution of each compound becomes increasingly uniform. Simultaneously, GHI decreases, reflecting the reduction in overall compositional variance as the blend homogenizes. It is interesting to note how GHI and DUI values clearly show the different blending behavior of the compounds in the mixture.

In contrast, Fig. 6b shows a more complex blending behavior in the pharmaceutical case. While DUI values for CA rise and stabilize, indicating uniform distribution, the DUI values for SSG and, more clearly for ASA, increase initially but start declining after approximately 300 s of blending. This decrease, coupled with a rising GHI trend, detects a de-mixing phenomenon, a known risk in prolonged blending where certain components may segregate due to differences in particle size, density, or electrostatic properties [49].

These results highlight the potential of SWiVIA to track both blending progression and deviations from ideal blending behavior in real time. The combined trends of DUI and GHI provide sensitive and interpretable indicators of both successful mixing and emergent faults such as over-blending or material stratification. Such insights are crucial for optimizing blending duration, improving formulation robustness, and ensuring consistent product quality. These tools not only support quality assurance of end products but also facilitate further development of closed-loop control systems for continuous manufacturing environments. Moreover, the use of SWiVIA in small-scale experiments provides a direct framework that can be extrapolated to industrial-scale blending units, supporting its integration into process analytical technology (PAT) strategies for real-time process control.

While variographic analysis and heterogeneity indices provide a powerful statistical framework for exploiting spatial information, other chemometric strategies explicitly incorporate spatial context. Texture-based descriptors, such as gray-level co-occurrence matrices (GLCM), wavelet features, morphological operations, and local-variance filters, have been coupled with multivariate models to capture structure across scales and improve classification/regression in industrial imaging [11, 50–53]. Among texture-based strategies, wavelet representations (2D/3D discrete wavelet transforms and wavelet packets) offer multi-scale spatial–spectral characterization and have been shown to enhance segmentation/classification and, in process-oriented applications, on-/in-line monitoring and quality control [54–58]. Beyond the concept of texture, frameworks combining spatial and spectral information (e.g., multiblock modeling or spatial regularization) further improve segmentation/classification and could be considered for process monitoring depending on the PAT objective [59–61].

## Conclusions

The combination of hyperspectral imaging and chemometrics has enormous potential to address many PAT challenges linked to the chemical and spatial definition of the qualitative, quantitative, and evolving nature of products and processes.

The spatial composition information captured by HSI serves as an exceptionally versatile input for chemometric approaches, enabling analysis at various spatial levels, from the overall image to individual objects, and even down to the pixel level for maximum spatial resolution.

Nowadays, with the high acquisition speed of pushbroom imaging systems and the low computational demand of many image-based soft sensors, real-time image-based PAT solutions have become a reality.

However, some aspects related to the use of HSI in industrial applications should be further investigated. The image data size is one of them, and the proposal and application of methodologies that may efficiently compress or select image essential information on-the-fly will become highly relevant to speed up even more online applications. Such a need may not be critical when pushbroom systems are used, since the amount of input data used for prediction in every frame is small; but if more powerful instrumentation comes into play, such as snapshot image systems, this aspect needs to be taken into account. Along the same direction, the storage of historical image information will also benefit from the use of smart data compression methodologies.

The other axis to be explored is image fusion. Although many industrial routine applications are easily solved using a single HSI platform, multimodal imaging has to be an option if required. Such a possibility requires further instrumental development of fast multimodal imaging platforms that can integrate different hyperspectral modalities in pushbroom mode for on-line applications. Even for off-line applications, multimodal platforms are still a costly solution. Recently, different chemometric approaches have been proposed to cope with the diversity of spatial resolutions and spectral diversity coming from multimodal imaging measurements, but these tools have not yet been tested in industrial applications.

This means that the combination of HSI and chemometrics is already implemented in the PAT toolbox, but the exciting challenges to be addressed will provide in the near future methodologies that may further improve the characterization of products and processes and will surely ensure the expansion of image-based solutions in the coming years.
